# Nanozymes: An emerging perspective in tumor diagnosis and treatment since the 21st century

**DOI:** 10.1097/MD.0000000000045475

**Published:** 2025-11-28

**Authors:** Qiong He, Xuejin Hu, Sanmao Liu

**Affiliations:** a Nanchong City Red Cross Center Blood Station, Nanchong City, Sichuan Province, China; b Respiratory Department, Bishan Hospital of Chongqing Medical University/Bishan Hospital of Chongqing, Bishan District, Chongqing City, China; c Division of Spinal Surgery, The First Affiliated Hospital of Guangxi Medical University, Nanning, Guangxi Zhuang Autonomous Region, People’s Republic of China.

**Keywords:** bibliometrics, biomaterials, cancer, data visualization, nanozymes

## Abstract

**Objective:**

This study aims to systematically explore the development pattern and influence of nanozyme technology in the field of oncology diagnosis and treatment through bibliometric analysis methods, while identifying which countries are the innovation drivers in this field.

**Methods:**

On October 16, 2024, a search of the Web of Science Core Collection (WoSCC) was conducted to review and analyze the application of nanozymes in oncology. A total of 977 studies published from 2007 to 2024 were retrieved and examined.

**Results:**

We found that global research interest and publication volume related to this topic continue to rise. China leads in international cooperation, publication volume, and citation frequency, highlighting its outstanding position in this field. The Chinese Academy of Sciences is the largest contributing institution in terms of publication volume. Notably, ACS Applied Materials & Interfaces and Advanced Healthcare Materials are the 2 most popular journals in this field. In authors, Yang, Piaoping leads with 25 articles, and Qu, Xiaogang is the most cited author. The keyword co-occurrence network reveals 3 hotspots: “nanozymes,” “photodynamic therapy,” and “peroxidase-like activity”; “cell-death,” “strategies,” “recent progress,” and “catalyst” are identified as trend topics for future exploration.

**Conclusion:**

Nanozymes hold significant promise for cancer diagnostics and therapy, with China, the United States, and Singapore leading the way in innovative research within this domain. Moving forward, research efforts should focus on fostering global collaboration and interdisciplinary synergy, innovate in the development of new nanozyme materials like SAzymes to improve catalytic performance and safety, investigate the potential role of nanozymes in cancer immunotherapy, and expedite the progression of nanozyme technologies from experimental settings to clinical use.

## 1. Introduction

Cancer remains one of the leading causes of death worldwide, and current treatment strategies, such as radiotherapy, chemotherapy, and surgical resection, often come with severe side effects. Emerging treatment methods, such as photodynamic therapy (PDT) and photothermal therapy (PTT), have become potential anti-cancer strategies due to their minimally invasive nature, high selectivity, and low toxicity. However, their clinical application is limited by drawbacks such as limited light penetration depth, tumor hypoxia, and the heat resistance of tumor cells.^[1–3]^ Therefore, the development of effective, easy-to-operate, and targeted treatment strategies has always been a key issue in the field of cancer prevention and treatment research. On this basis, the new concept of “nano-catalytic medicine” has emerged, and nanozymes have been rapidly applied in the diagnosis and treatment of cancer.

Nanozymes, also known as nanozyme catalysts or enzyme-mimicking nanoparticles, are primarily composed of metal nanoparticles, oxide nanoparticles, etc, and they can mimic the catalytic actions of natural enzymes, including oxidoreductase, peroxidase (POD), and protease activities.^[4–8]^ They were 1st discovered and reported in 2007 by a team led by Academician Yan Xiyun of the Chinese Academy of Sciences.^[9–11]^ This discovery marked an important breakthrough of nanomaterials in the field of biocatalysis and has had a profound impact on medical research.^[12]^ Nanozymes have shown broad application prospects in tumor diagnosis and treatment due to their tumor-targeting ability, low toxicity, and capacity to locally generate high concentrations of reactive oxygen species.^[13–18]^ Over the past decade, nanozymes have provided new strategies for cancer treatment with their unique catalytic properties, stability, and ease of functionalization. As a new type of artificial enzyme-mimicking nanomaterial, they have attracted widespread attention from researchers globally.

**Figure 1.**
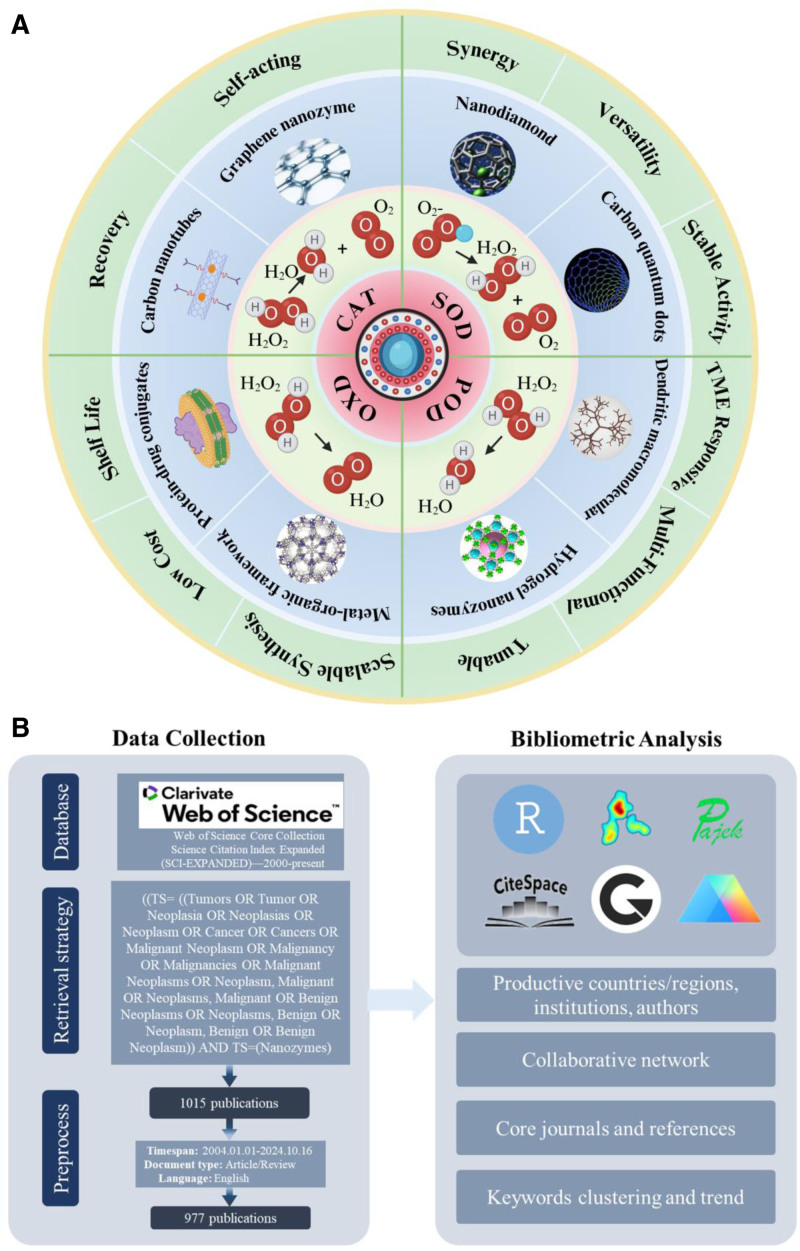
(A) Currently, the enzyme-catalyzed reactions exhibited by the 4 common types of tumor nanozymes, namely catalase (CAT), superoxide dismutase (SOD), peroxidase (POD), and oxidase (OXD), as well as common nanometal materials and the ideal characteristics of nanozymes. (B) Retrieval and analysis flowchart.

In recent years, significant achievements have been made in the research of nanozymes. Common nanozymes that have successfully mimicked biological catalytic activities include POD-mimicking nanozymes, oxidase-mimicking nanozymes, superoxide dismutase-mimicking nanozymes, and catalase (CAT)-mimicking nanozymes, among which POD-mimicking nanozymes are most widely used in the oncology field. The main nanomaterials can be classified based on their composition into metal, alloy, and metal oxide nanoparticles, core–shell composite materials, nanozymes based on different carbon allotropes (e.g., carbon nanotubes, graphene, nanodiamonds, carbon quantum dots, dendrimers, hydrogels, ordered porous materials such as metal-organic frameworks (MOFs), and protein–drug conjugates) (Fig. 1A).^[19–28]^ Moreover, improvements in the synthesis methods, structural characterization, and biocompatibility of nanozymes have greatly promoted their application in oncology.^[29–31]^ Xue et al^[32]^ and Liu et al^[33]^ have improved the stability and catalytic efficiency of nanozymes through surface modification and size control, enhancing the synthesis and modification technologies of nanozymes. Li et al^[34]^ and Keyvani et al^[35]^ pointed out that nanozymes can be used as contrast agents to improve imaging resolution or as biomarker detectors to enhance detection sensitivity, greatly aiding in the diagnosis of cancer. Yang et al^[36]^ and Lu et al^[37]^ indicated that nanozymes exhibit excellent performance in tumor-targeted therapy, enabling precise treatment of tumor tissues through active or passive targeting strategies. Chen et al^[38]^ and Chen et al^[39]^ combined nanozymes with traditional treatment methods such as chemotherapy, radiotherapy, and PTT, providing new combined treatment strategies for cancer therapy. To our knowledge, a bibliometric study on the application of nanozymes in the oncology field has not yet been conducted.

Therefore, the research focuses on how nanozymes, through their unique catalytic activities and biological functions, are driving innovative changes in tumor diagnostic and therapeutic strategies, thereby redefining the technological development direction and clinical application prospects in the field of tumor diagnosis and treatment, while also identifying which countries are the innovation drivers in this field. Additionally, this paper is expected to provide a useful reference for further research on nanozymes in oncology and offer decision support for researchers and policymakers in related fields.

## 2. Materials and methods

### 2.1. Search strategy

On October 16, 2024, a literature search was conducted using the Web of Science Core Collection (WoSCC) database to retrieve relevant publications. The following search parameters were applied: ((TS= ((Tumors OR Tumor OR Neoplasia OR Neoplasias OR Neoplasm OR Cancer OR Cancers OR Malignant Neoplasm OR Malignancy OR Malignancies OR Malignant Neoplasms OR Neoplasm, Malignant OR Neoplasms, Malignant OR Benign Neoplasms OR Neoplasms, Benign OR Neoplasm, Benign OR Benign Neoplasm)) AND TS = (Nanozymes) AND DT = (Article OR Review)) AND LA = (English)). The search included articles and reviews that mentioned “nanozymes” and “tumor” or their synonyms in the title, abstract, or keywords. Only documents written in English and published between January 1, 2000, and October 16, 2024, were considered, while case reports, conference abstracts, editorial materials, and other document types published before January 1, 2000, were excluded (Fig. 1B).

### 2.2. Methods

A bibliometric analysis was conducted using various software tools and packages. The software tools and packages used included HisCite (version 12.03.17), VOSviewer (version 1.6.18), CiteSpace (version 6.1.R3), and the R-based bibliometrix package (version 3.2.1). HisCite was used to calculate the total number of publications and citations based on multiple criteria, including the publishing country, institution, and author. Closeness centrality measures the average distance of a node to all other nodes in the network, with higher values indicating that the node can influence other nodes in the network more rapidly. Betweenness centrality measures the frequency with which a node appears on the shortest paths between other nodes in the network, with higher values indicating that the node plays a “bridge” role, possessing a stronger ability to control information flow.^[40,41]^ This article will utilize these 2 network analysis indicators to quantify the influence and importance of institutional and author collaborations. Additionally, HisCite was used to determine the top 10 most cited papers in the field. The number of papers published each year was determined using HisCite, and the data were visualized using the ggplot2 package (version 3.3.6) in the R programming language. VOSviewer was used to identify the top 10 most frequently occurring keywords, analyze bibliometric coupling within journals, and generate a visual overlay of journals related to this direction. CiteSpace was used to assess the centrality of collaboration among countries/regions, institutions, and authors. Furthermore, the bibliometrix package was used as an alternative method for trend topic detection. The package was also used to construct a visualization network illustrating the number of publications and collaboration relationships. Overall, these software tools and packages made a comprehensive bibliometric analysis possible, providing insights into publication and citation patterns, keyword occurrence, collaboration networks, and research trends of nanozymes in the oncology field.

## 3. Results

### 3.1. Overview

**Figure 2.**
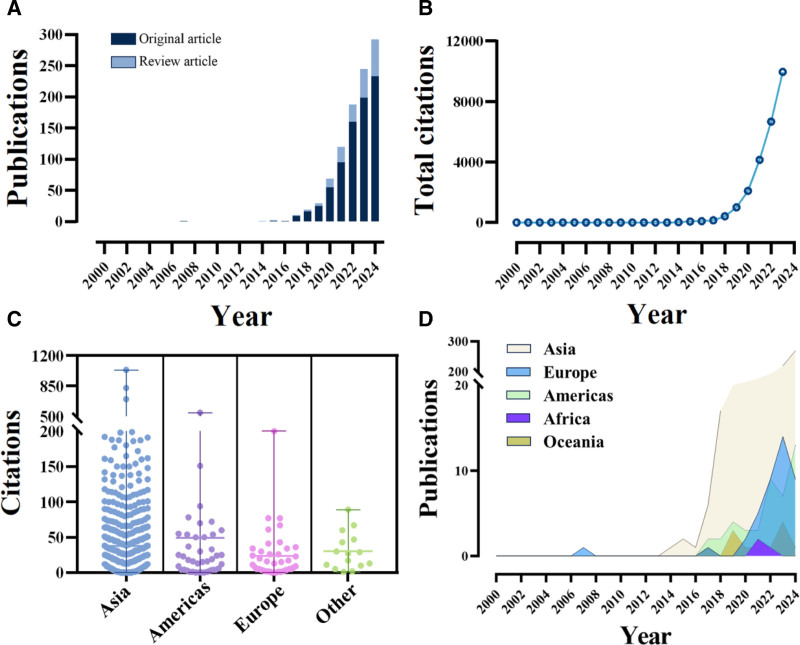
The yearly quantity, citations of publications pertaining to tumor nanozymes and country collaboration clustering. (A) The yearly quantity pertaining to tumor nanozymes. (B) The yearly citations pertaining to tumor nanozymes. (C) Citation counts by continent. Scatterplot (n): Asia = 873, Americas = 43, Europe = 41, Other (Oceania and Africa) = 15; Error bars: Asia = 37.46 citations, Americas = 49.26 citations, Europe = 23.37 citations, Other = 30.07 citations. Statistical analysis using non-parametric testing (Mann–Whitney *U* rank sum test) revealed significant differences between the Americas and Europe (*P* < .01**) as well as between the Americas and Other (*P* < .05*). (Note: * *P* < .05, ** *P* < .01, *** *P* < .001.). (D) The yearly publications to each continent.

A thorough exploration of the WoSCC database yielded 977 publications related to nanozymes and cancer. The search, covering the period from January 1, 2000, to October 16, 2024, resulted in 977 publications closely related to this field. Of these publications, 795 were classified as original articles, while 182 were classified as review articles. Notably, although publications on the application of nanozymes in cancer started to appear in 2007, the number of publications has rapidly increased since then; the total citation count also showed a clear upward trend (Fig. 2A, B). It is noteworthy that original articles consistently outnumbered review articles. These published articles have accumulated a total of 35,235 citations, with an average of 36.06 citations per article, indicating considerable academic significance.

### 3.2. Major countries/regions

**Figure 3.**
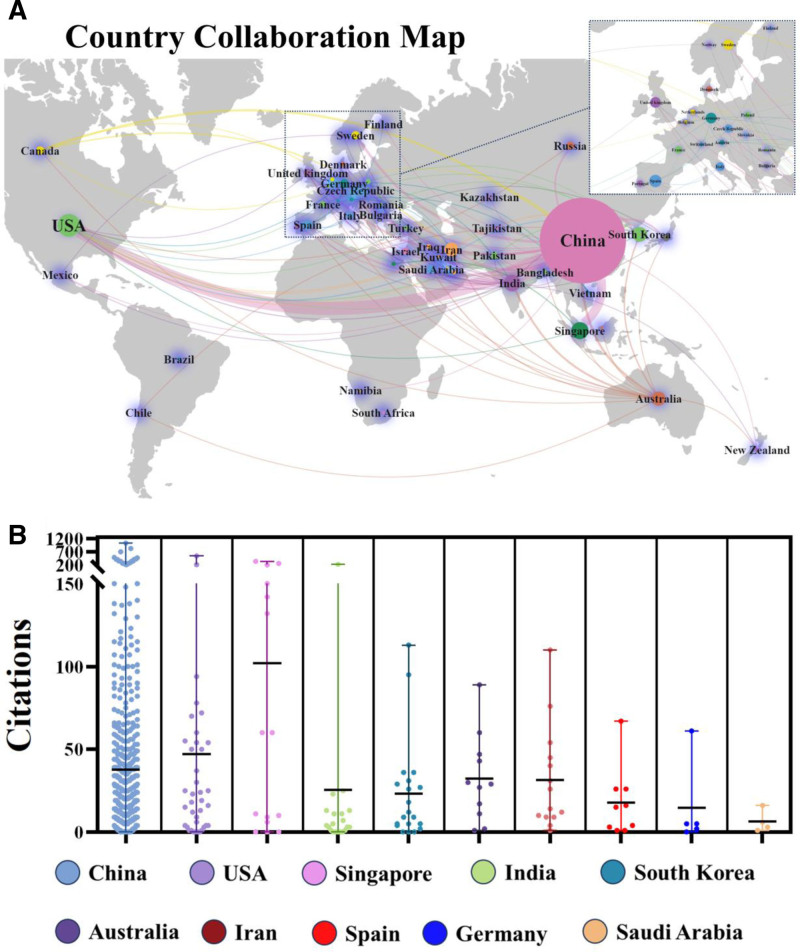
Country collaboration map and literature citation beeswarm diagram. (A) Country collaboration map. (B) Citation counts for the top 10 countries with the highest publication output. The number of publications (n) and average citation counts for each country/region are as follows: China (681, 36.91), USA (60, 41.57), Singapore (34, 76.50), India (32, 22.72), South Korea (25, 28.84), Australia (23, 45.83), Iran (22, 30.77), Spain (12, 18.17), Germany (9, 22.11), and Saudi Arabia (9, 25.11). Statistical analysis using the Kruskal–Wallis test (overall difference) revealed significant differences in the average citation counts of nanozyme papers among different countries (*P* < .01**).

From January 1, 2000, to October 16, 2024, academic papers on cancer nanozymes were published in 49 countries across 6 continents. Notably, there was significant collaboration between North America, Asia, and Oceania (Fig. 3A). This study focused on the top 10 countries by the number of publications on tumor nanozymes. China led with 681 articles (69.70%), followed by the United States (60 articles, 6.14%), Singapore (34 articles, 3.48%), India (32 articles, 3.27%), and South Korea (25 articles, 2.55%). Notably, publications from the United States received the highest total citations (31,518) and average citations (36.91), while Singapore had the highest average citations (76.50) with a total of 2601 citations (Table 1; Fig. 2C, D; Fig. 3B).

**Table 1:** The top 10 countries/regions with the highest productivity in the field of tumor nanozymes.

Rank	Country	Publications n (%)	Total citations	Average citations	Collaborative centrality
1	China	854 (87.41%)	31,518	36.91	0.93
2	USA	60 (6.14%)	2494	41.57	0.15
3	Singapore	34 (3.48%)	2601	76.50	0.01
4	India	32 (3.27%)	727	22.72	0.17
5	South Korea	25 (2.55%)	721	28.84	0.11
6	Australia	23 (2.35%)	1054	45.83	0.21
7	Iran	22 (2.25%)	677	30.77	0.22
8	Spain	12 (1.22%)	218	18.17	0.02
9	Germany	9 (0.92%)	199	22.11	0.11
10	Saudi Arabia	9 (0.92%)	226	25.11	0.06

*Note*: collaborative centrality is a concept in network analysis that measures the importance of a node (such as an author, institution, or country) in terms of its collaborative connections with other nodes in a network.

### 3.3. Active institutions and authors

**Figure 4.**
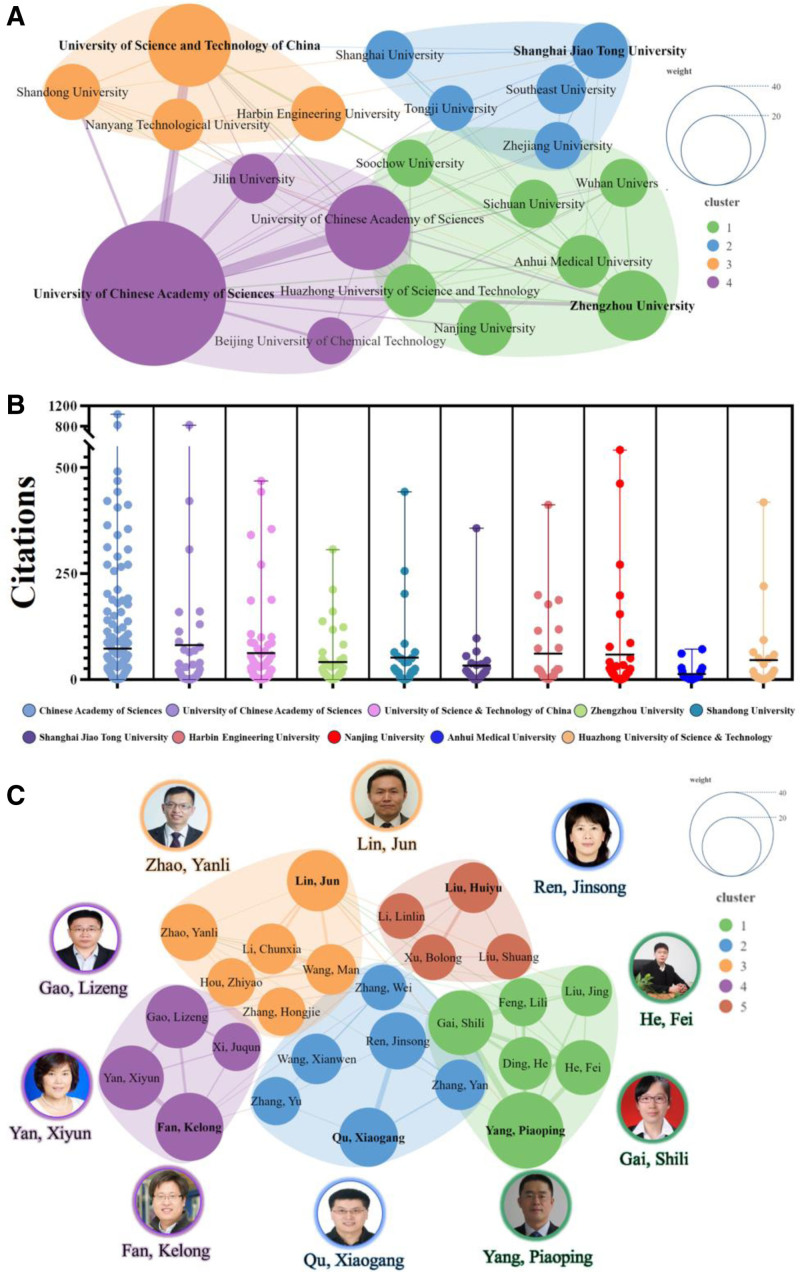
Collaborative clustering of institutions and authors. (A) Collaborative clustering of institutions. (B) Citation counts for the top 10 institutions with the highest publication output. The number of publications (n) and average citation counts for each institution are as follows: Chinese Academy of Sciences (180, 74.58), University of the Chinese Academy of Sciences (n = 62, 102.90), University of Science and Technology of China (59, 61.78), Zhengzhou University (42, 40.24), Shandong University (27, 55.63), Shanghai Jiao Tong University (27, 32.33), Harbin Engineering University (26, 59.15), Nanjing University (26, 78.62), Anhui Medical University (24, 12.79), and Huazhong University of Science and Technology (24, 44.54). Statistical analysis using the Kruskal–Wallis test (overall difference) revealed significant differences in the average citation counts of nanozyme papers among different institutions. (C) Collaborative clustering of authors.

The comprehensive investigation revealed that the 977 publications were associated with 854 institutions in 49 countries/regions and were authored by 5290 individuals. Among these institutions, the Chinese Academy of Sciences topped the list with 180 publications (18.42%). It was followed by the University of the Chinese Academy of Sciences (62 publications, 6.34%) and the University of Science and Technology of China (59 publications, 6.03%). Zhengzhou University was notable for 42 publications (4.29%), and Shandong University published 27 notable papers (2.76%). Proudly, all of the top 10 institutions were located in China; Simultaneously, the Chinese Academy of Sciences possesses the highest closeness centrality and betweenness centrality (0.9375, 0.3005), with Zhengzhou University (0.7143, 0.0792) and University of Chinese Academy of Sciences (0.6977, 0.0591) also demonstrating considerable closeness centrality and betweenness centrality (Table 2). Twenty academic institutions with more than 18 publications were included in the cluster analysis, and the visualization chart illustrated the degree of collaboration among these institutions (Fig. 4A, B). The honor of the most productive author went to Yang, Piaoping, who authored 25 works (2.55%), followed by Fan, Kelong with 20 commendable papers (2.47%), and Gai, Shili with 18 notable contributions (1.84%); notably, 9 out of the top 10 authors were from China, with China’s Lin, Jun having the highest H-index^[42]^ of (123). Simultaneously, Professor Gao Lizeng and Professor Fan Kelong possess the highest closeness centrality (0.2828) and betweenness centrality (0.1235), respectively, with Professor Yang Piaoping and Professor Zhao Yanli also exhibiting considerable closeness centrality (0.2725) and betweenness centrality (0.0665) (Table 3). Additionally, 30 authors with more than 9 publications were included in the cluster analysis, of which 26 were in collaboration, and the visualization chart showed the degree of collaboration among these authors (Fig. 4C).

**Table 2:** The top 10 productive institutions in the field of tumor nanozymes.

Rank	Institution	Country	Publications n (%)	Total citation	Average citation	Closeness centrality	Betweenness centrality
1	Chinese Academy of Sciences	China	180 (18.4%)	13,425	74.58	0.9375	0.3005
2	University of the Chinese Academy of Sciences	China	62 (6.34%)	6380	102.90	0.6977	0.0591
3	University of Science and Technology of China	China	59 (6.03%)	3645	61.78	0.6522	0.0378
4	Zhengzhou University	China	42 (4.29%)	1690	40.24	0.7143	0.0792
5	Shandong University	China	27 (2.76%)	1502	55.63	0.6000	0.0135
6	Shanghai Jiao Tong University	China	27 (2.76%)	873	32.33	0.6122	0.0272
7	Harbin Engineering University	China	26 (2.66%)	1538	59.15	0.5556	0.0021
8	Nanjing University	China	26 (2.66%)	2044	78.62	0.5455	0.0028
9	Anhui Medical University	China	24 (2.45%)	307	12.79	0.5660	0.0223
10	Huazhong University of Science and Technology	China	24 (2.45%)	1069	44.54	0.6383	0.0190

**Table 3:** Top 10 productive authors in the field of tumor nanozymes.

Rank	Author	Institution	Country	Publications n (%)	Total citation	Average citation	*H*-index	Closeness centrality	Betweenness centrality
1	Yang, Piaoping	Harbin Engineering University	China	25 (2.55%)	1535	61.40	85	0.2725	0.0473
2	Fan, Kelong	Zhengzhou University	China	20 (2.04%)	2605	130.25	53	0.2812	0.1235
3	Gai, Shili	Harbin Engineering University	China	18 (1.84%)	1283	71.28	72	0.2581	0.0085
4	Yan, Xiyun	Zhengzhou University	China	18 (1.84%)	2402	133.44	58	0.2722	0.0542
5	Gao, Lizeng	University of Chinese Academy of Sciences	China	17 (0.17%)	2345	137.94	31	0.2828	0.0580
6	Lin, Jun	University of Science and Technology of China	China	16 (0.16%)	2378	148.63	123	0.2496	0.0312
7	Qu, Xiaogang	University of Science and Technology of China	China	15 (0.15%)	3248	216.53	114	0.2389	0.0084
8	Ren, Jinsong	University of Science and Technology of China	China	14 (0.14%)	3143	224.50	108	0.2389	0.0084
9	Zhao, Yanli	Nanyang Technological University	Singapore	14 (0.14%)	1413	100.93	111	0.2615	0.0665
10	He, Fei	Harbin Engineering University	China	13 (0.14%)	1172	90.15	72	0.2523	0.0309

*Note*: *H*-index: indicates the impact of a scientist’s published paper, and the 2 are positively correlated.

### 3.4. Core journals and references

**Figure 5.**
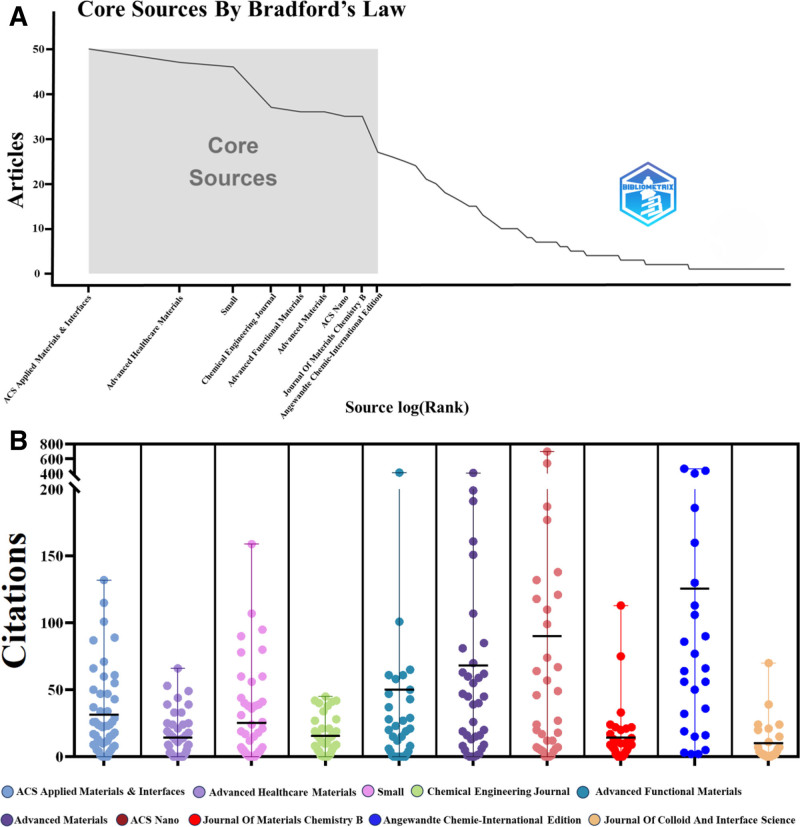
Analysis of journals. (A) Core journals in the field as delineated by Bradford Law. (B) Citation counts for the top 10 journals with the highest publication output. The number of publications (n) and average citation counts for each journal are as follows: ACS Applied Materials & Interfaces (50, 30.32), Advanced Healthcare Materials (47, 14.13), Small (46, 27.43), Chemical Engineering Journal (37, 15.46), Advanced Functional Materials (36, 49.00), Advanced Materials (36, 70.44), ACS Nano (n35, 8.03), Journal Of Materials Chemistry B (35, 14.17), Angewandte Chemie-International Edition (27, 122.37), and Journal Of Colloid And Interface Science (26, 10.12). Statistical analysis using the Kruskal–Wallis test (overall difference) revealed significant differences: *P*-value < 0.01, indicating significant differences in the average citation counts of nanozyme papers among these journals.

A total of 198 reputable journals contributed to the dissemination of research articles related to tumor nanozymes. Among these journals, ACS Applied Materials & Interfaces stood out with 50 articles (5.11%), making it the journal with the highest number of publications. It was followed by Advanced Healthcare Materials (47 articles, 4.81%), Small (46 articles, 4.70%), Chemical Engineering Journal (37 articles, 3.78%), and Advanced Functional Materials (36 articles, 3.68%). Notably, Angewandte Chemie-International Edition had the highest total citations (3304) and the highest average citation rate per article (122.37) (Table 4). The Bradford Law, an important principle in bibliometrics, discusses the distribution of scientific literature in a specific field and suggests that a small number of core sources or journals make significant contributions to all research published in that field. The top 9 journals and their citation counts in the tumor nanozyme field are shown in Figure 5A, B. Additionally, we summarized the top 10 most cited papers^[43–52]^ (Table 5). A careful review of these articles revealed that the core references focus on the diagnosis and treatment of tumors using nanozymes.

**Table 4:** Ten core journals with the highest citations in the field of tumor nanozymes.

Rank	Journal	Publications n (%)	Total citations	Average citations	2023 JCR category quartile	2023IF
1	ACS Applied Materials & Interfaces	50 (5.11%)	1516	30.32	Q1	8.3
2	Advanced Healthcare Materials	47 (4.81%)	664	14.13	Q1	10.0
3	Small	46 (4.70%)	1262	27.43	Q1	13.0
4	Chemical Engineering Journal	37 (3.78%)	572	15.46	Q1	13.3
5	Advanced Functional Materials	36 (3.68%)	1764	49.00	Q1	18.5
6	Advanced Materials	36 (3.68%)	2536	70.44	Q1	27.4
7	ACS Nano	35 (3.58%)	3081	88.03	Q1	15.8
8	Journal Of Materials Chemistry B	35 (3.58%)	496	14.17	Q1	6.1
9	Angewandte Chemie-International Edition	27 (2.76%)	3304	122.37	Q1	16.1
10	Journal Of Colloid And Interface Science	26 (2.66%)	263	10.12	Q1	9.4

*Note*: JCR: Journal Citation Reports, published by the Science Information Institute (SI) in the United States, is the most fundamental, comprehensive, and unique tool for evaluating scientific and technical journal papers in the world; IF: impact factor, which refers to the total number of citations of articles published in a journal in the 2 years prior to a given year divided by the total number of articles published by the journal in those 2 years.

**Table 5:** Ten core literatures with the highest citations in the field of tumor nanozymes.

Rank	First author	Title	Journal	Type	Year of publication	Total citations
1	Lin, Youhui	Catalytically Active Nanomaterials: A Promising Candidate for Artificial Enzymes	Accounts of Chemical Research	Review	2014	1035
2	Fan, Kelong	In Vivo Guiding Nitrogen-Doped Carbon Nanozyme For Tumor Catalytic Therapy	Nature Communications	Article	2018	828
3	Zhang, Yan	Nanozyme Decorated Metal-Organic Frameworks for Enhanced Photodynamic Therapy	ACS Nano	Article	2018	699
4	Hu, Yihui	Surface-Enhanced Raman Scattering Active Gold Nanoparticles with Enzyme-Mimicking Activities for Measuring Glucose and Lactate in Living Tissues	ACS Nano	Article	2017	542
5	Gao, Shanshan	Nanocatalytic Tumor Therapy by Biomimetic Dual-Inorganic Nanozyme Catalyzed Cascade Reaction	Advanced Science	Article	2019	491
6	Yao, Jia	ROS scavenging Mn_3_O_4_ nanozymes for in vivo anti-inflammation	Chemical Science	Article	2018	462
7	Sun, Hanjun	Carbon Nanozymes: Enzymatic Properties, Catalytic Mechanism, and Applications	Angewandte Chemie-International Edition	Review	2018	469
8	Chang, Mengyu	Single-Atom Pd Nanozyme for Ferroptosis-Boosted Mild-Temperature Photothermal Therapy	Angewandte Chemie-International Edition	Article	2021	443
9	Dong, Shuming	GSH-Depleted Nanozymes with Hyperthermia-Enhanced Dual Enzyme-Mimic Activities for Tumor Nanocatalytic Therapy	Advanced Functional Materials	Article	2020	418
10	Gao, Lizeng	Iron Oxide Nanozyme: A Multifunctional Enzyme Mimetic for Biomedical Applications	Theranostics	Review	2017	421

ROS = Reactive oxygen species.

### 3.5. Keyword analysis

**Figure 6.**
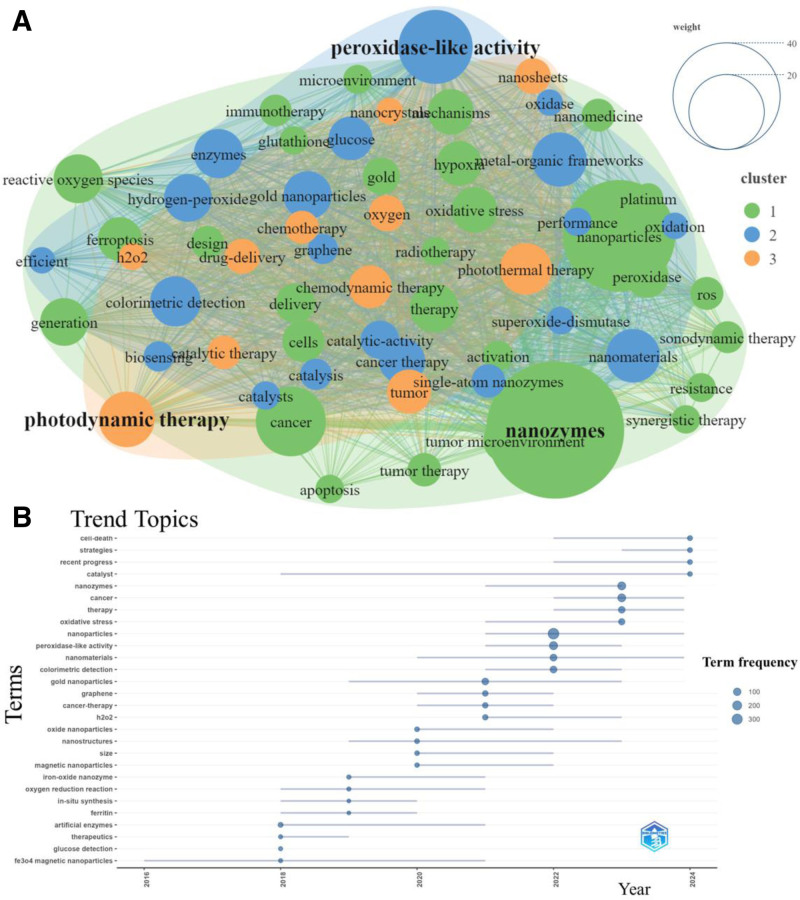
Analysis of keywords. (A) Cluster map of 60 keywords with frequency of occurrence > 20. (B) Analysis of trend topics.

**Figure 7.**
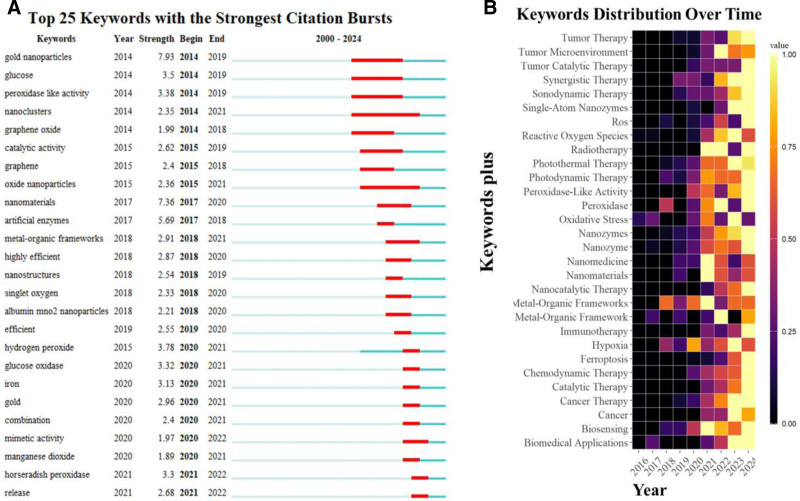
(A) The top 25 most co-cited keywords. The years between the beginnings and endings represent periods in which the keywords were more influential, with light green years indicating that the keywords had not yet appeared, dark green years indicating that the keywords were less influential, and red years indicating that the keywords were more influential. (B) Keyword heat map.

An analysis of the co-occurrence of 60 keywords with a frequency of more than 20 revealed 3 distinct clusters. The cluster including “nanozymes,” “photodynamic therapy” and “peroxidase-like activity” had the highest frequency (Fig. 6A). A check of trend topics from 2016 to 2024 revealed that “cell-death,” “strategies,” “recent progress,” and “catalyst” are the focus of future research efforts (Fig. 6B). This study also used keyword burst detection technology to identify the top 25 keywords with the strongest bursts and corresponding burst durations. Notably, “horseradish peroxidase (HRP)” and “release” were the most influential keywords in 2022. “Nanoclusters” was identified as the keyword with the strongest burst, with a duration from 2014 to 2021 (Fig. 7A). Additionally, this compilation also showcases the popular keywords used each year (Fig. 7B).

## 4. Discussion

This study represents the 1st comprehensive bibliometric analysis of tumor nanozyme research globally, providing valuable insights for beginners to understand the historical development and trends of the field. The results of this study show that the number of publications and citations in tumor nanozyme research has steadily increased over the past 20 years, highlighting the increasing importance of the field to scholars, this is largely consistent with the findings of Wu et al^[53]^ on the use of nanozymes in combination with sonodynamic therapy for tumor treatment. In recent years, there has been a rapid growth in related research, indicating that the role of nanozymes in tumor prevention and treatment is becoming increasingly prominent. Notably, China holds a dominant lead in terms of the total number of publications and total citations. Although the United States ranks 2nd in the number of publications, it maintains close ties with China in this field. Simultaneously, Singapore ranks 3rd in the total number of publications but has the highest average citations and the 2nd-highest total citations (Fig. 2, Table 1). These results suggest that China, the United States, and Singapore are the innovation drivers in the field of tumor nanozymes.

At the institutional level, the top 10 institutions are all located in China, with the Chinese Academy of Sciences in the lead. It ranks 1st in both total citations and average citations. In terms of centrality, the Chinese Academy of Sciences exhibits the highest closeness centrality and betweenness centrality (0.9375, 0.3005), indicating its highly central position in the network, enabling rapid information exchange with other nodes, and playing a significant role as a bridge in information flow. Additionally, Zhengzhou University and University of Chinese Academy of Sciences also demonstrate considerable closeness centrality and betweenness centrality, suggesting their influence in network information transmission. Previous studies have also found that Chinese Academy of Sciences has outstanding performance in nanozyme research for tumor treatment, ranking 1st globally in terms of total citations and average citations per article.^[54,55]^ Chinese institutions have focused their research on the design and multifunctionalization of nanozymes, such as the development of iron-based, platinum-based, and single-atom nanozymes. These nanozymes mimic the activities of POD, oxidase, and CAT, inducing ferroptosis, apoptosis, and immunogenic cell death.^[56–58]^ These studies not only enhance catalytic efficiency but also improve the tumor targeting and biocompatibility of nanozymes through structural engineering, such as hollow structures and high-entropy design, solidifying China’s leading position in this field. The current collaborative network in nanozyme research exhibits a pronounced regionalization trend, with close cooperation among institutions in East Asia (particularly China), North America, Europe, and Australia, but weaker collaboration with other regions (such as South America and Africa).^[59,60]^ This imbalance may stem from differences in the distribution of scientific resources, policy support, and academic exchange channels. For instance, Chinese institutions have joint research with European and American teams in exploring nanozyme mechanisms (such as catalytic activity and tumor microenvironment [TME] responsiveness), but intercontinental collaboration mostly remains at the level of theoretical discussion, lacking deep integration in clinical translation.^[61–63]^ Previous literature has also shown that the limitations of regional cooperation may delay the clinical application of nanozymes. On one hand, the heterogeneity of the TME requires nanozyme design to consider the characteristics of different populations and tumor types, and the sample limitations of a single region may lead to insufficient generalizability of research conclusions.^[64,65]^ On the other hand, international cooperation can help integrate strengths from multiple regions (such as imaging technologies in Europe and immunotherapy experiences in North America), promoting innovation in multimodal diagnosis and treatment (such as photoacoustic imaging and immunosynergistic therapy) with nanozymes.^[63,66,67]^ The lack of global collaboration may result in redundant resource investment and a disconnection between theory and application.^[68,69]^ In summary, as an emerging tool for tumor treatment, the development of nanozymes heavily relies on international collaboration and resource integration. Although Chinese institutions lead in basic research, the imbalance in the global collaboration network may limit the potential for clinical translation. Future efforts should focus on establishing transnational platforms, sharing data resources, and deepening interdisciplinary cooperation to promote the widespread application of nanozymes in precision tumor therapy, ultimately achieving the transition from laboratory innovation to clinical benefits. Specific measures include: establishing transnational academic alliances to conduct multicentric preclinical studies and validate the universality of nanozymes in different TME models^[60,70]^; data sharing and standardization to build databases of nanozyme catalytic activities and TME response standards, enhancing the comparability and reproducibility of results^[64,68]^; interdisciplinary integration to merge expertise in materials science, immunology, and clinical medicine, developing intelligent nanozymes (such as pH-responsive, photocontrolled catalysis) to improve treatment precision.^[71,72]^

Regarding authors, 9 out of the top 10 authors with the most publications are from China, with Yang, Piaoping publishing the most articles (25). Professor Yang is a renowned scholar in the field of nanomedicine delivery systems, with his primary research focus encompassing the design and fabrication of nanocarrier materials, the construction of targeted drug delivery systems, and the application of nanomedicines in tumor therapy. He is dedicated to developing various delivery systems, such as liposomes, polymer nanoparticles, and inorganic nanomaterials, to enhance the targeting and bioavailability of drugs while reducing toxic side effects. These studies provide new strategies for precision tumor therapy and propel the clinical translation process of nanomedicine.^[73,74]^ Additionally, Lin, Jun has an impressive *H*-index of 123, his team has developed a variety of novel nanozyme materials, including MOFs, metal oxide nanoparticles, and carbon-based nanozymes. These materials possess enzyme-mimetic catalytic activities and hold broad application prospects in the biomedical field.^[75,76]^ Another renowned author, Zhao, Yanli from Singapore, ranks ninth in publications (14), but has a higher *H*-index score of 111. Professor Zhao is dedicated to designing and synthesizing nanozyme materials with high catalytic activity and stability, particularly making significant progress in noble metal nanozymes, magnetic nanozymes, and carbon-based nanozymes. These materials possess extensive potential applications in biomedical and environmental fields.^[77,78]^ The analysis of author collaboration shows poor research relationships between authors, indicating limited academic connections and communication. Notably, Yang, Piaoping emerged as a central figure in collaborations, but the scope is limited to China; Simultaneously, Professor Gao Lizeng possesses the highest closeness centrality (0.2828), indicating a central position within the network, enabling efficient information dissemination with the shortest paths to all other authors. This reflects his extensive direct collaborative relationships in the field, acting as an “information hub.” Professor Fan Kelong has the highest betweenness centrality (0.1235), signifying a critical role in bridging different collaborative groups. When communication between network groups is necessary, Professor Fan often serves as a necessary conduit, demonstrating his capability for cross-disciplinary and inter-institutional collaboration, and playing an irreplaceable role in facilitating academic exchange and information flow. Therefore, authors from different countries and institutions should strive to strengthen collaboration to collectively improve the research standards in the field of tumor nanozymes.

The citation patterns, impact factors, and publication practices of academic journals are crucial indicators for assessing research impact and the efficiency of scholarly communication.^[79–81]^ Therefore, we conducted a thorough investigation of the journals associated with the publications to reveal further insights. The results indicate that journals such as ACS Applied Materials & Interfaces, Advanced Healthcare Materials, and Small rank among the top 3 in terms of the number of publications and citations. Moreover, Angewandte Chemie-International Edition leads in terms of average citations, and all these journals have an impact factor exceeding 8. Researchers seeking to publish high-quality work are expected to find these 10 journals as valuable resources.

Furthermore, most of the top 10 most cited papers are related to the diagnosis and treatment of tumors using nanozymes, indicating that these areas have always attracted the attention of researchers. For example, in 2018, Zhang et al^[45]^ introduced a method for treating tumors using nanozyme-modified MOFs to enhance PDT; in 2019, Gao et al^[47]^ used bionic dual-inorganic nanozymes to catalyze cascade reactions for nanocatalytic tumor therapy. These highly cited publications can be considered valuable and influential research in the field, providing a basis for new researchers to explore further. However, as the field evolves, the limitations of using nanozymes as a standalone treatment for tumors have become apparent, and the combination of nanozymes with other therapies has gained consensus. For instance, Bi₂Se₃ nanodisks inhibit the inflammatory response induced by PTT through their multi-enzyme activity, achieving non-inflammatory hyperthermia.^[82]^ Mn/Fe-MIL-101/CuS@FA tri-metal nanozymes suppress tumor recurrence and metastasis through photothermal-enhanced chemodynamic therapy and immunogenic cell death effects.^[83]^ Nanozymes can also regulate macrophage polarization (e.g., CuFeSe₂-AMD3100-Gem), reversing the immunosuppressive microenvironment.^[84,85]^

Previous studies have demonstrated that nanozymes can combat tumor cells through photothermal effects and photodynamic regulation of POD-like activity.^[57,86]^ For example, Yim et al^[87]^ demonstrated that POD-like activity of nanozymes and PDT can jointly induce apoptosis in cancer cells by generating reactive oxygen species. In this study, the examination of annual trend topics over the past 20 years highlighted research trends such as “cell-death,” “strategies,” “recent progress,” and “catalyst” in 2024, which may indicate the research frontiers for the coming years.^[88–90]^ Meanwhile, the burst detection algorithm helped reveal the popularity of hot keywords during specific periods and identified active discussion topics. As shown in Figure 7A, popular keywords in recent years have mainly focused on “HRP,” “release,” and “nanoclusters.” These findings indicate an inseparable connection between “HRP,” “release,” and “nanoclusters.” In fact, single-atom catalysts have attracted attention in the past decade because they maximize the utilization of active sites and help understand product distribution in certain catalytic reactions. Recently, this concept has been extended to single-atom nanozymes, which are used to simulate natural enzymes commonly used in bioanalytical applications, such as HRP. This is a major simulation direction for tumor nanozymes in recent years and is closely related to the diagnosis and treatment of tumors. Furthermore, in recent years, nanoclusters have been recognized as perfect mimics of HRP, playing an important role in the detection of glucose and glutathione, among others.^[91–95]^

Nanozymes have provided new options for the diagnosis and treatment of tumors and are playing an increasingly important role in the field of human tumor research.^[54,65,96–98]^ Recently, some excellent retrospective studies have focused on the progress of tumor nanozyme research from different perspectives. Fan et al summarized the dual-functional catalytic activity of transition metal catalysts, the design of nanozymes using machine learning, and the multi-faceted nanocatalytic therapy for anti-tumor treatment.^[99–101]^ Huang et al^[102]^ highlighted the progress of nanozymes before 2018, focusing on classification, catalytic mechanisms, activity regulation, and applications, while recent developments such as machine learning and theoretical calculations were not included. Zhang et al^[103]^ focused on the biomedical applications of nanozymes, including cancer diagnosis and treatment. Ding et al^[104]^ described the strategies for activity regulation in nanozyme biomedical applications (i.e., doping, vacancy, modification, size, and morphology). Yang et al^[105]^ introduced the activity of nanozymes and their biomedical applications.

Currently, the application of nanozymes in tumor immunotherapy is still in its infancy. Yang et al^[106]^ have reported a pH- and H₂O₂-responsive manganese-based nanozyme that exhibits POD- and CAT-like activities, significantly enhancing the activity of cytotoxic T lymphocytes. Future research can explore how nanozymes regulate the function of immune cells in the TME, such as by modulating macrophage polarization, enhancing T cell infiltration, or affecting the expression of immune checkpoint molecules, thereby improving the efficacy of immunotherapy. Single-atom nanozymes (SAzymes) are a class of emerging nanocatalytic materials whose active sites consist of atomically dispersed metal centers, possessing nearly 100% atomic utilization and well-defined geometric/electronic structures.^[107,108]^ Currently, the application of SAzymes in the field of tumors is limited. Future research can investigate the potential applications of SAzymes in tumor catalytic therapy, biosensing, and anti-infection treatment.

As shown in Figure 2, the 1st study on nanozymes was published in 2007. However, we set the starting year of our literature search to 2000 for the following reasons: firstly, as an emerging field, the theoretical foundation and technological development of nanozymes may originate from earlier nanotechnology and enzymology research. By setting the search start year to 2000, we can comprehensively capture the relevant research background preceding the conception of nanozymes, including early explorations of nanomaterial catalytic properties, preliminary studies on enzyme-mimicking activities, and the development of related characterization techniques. For instance, in 2004, the term “nanozyme” was 1st used to describe the transphosphorylation reaction activity of gold nanoparticles modified with aziridine.^[109]^ Secondly, although the types of nanozyme articles that appeared in 2007 were primarily the articles and reviews we required, during our literature review, we found that before 2007, there were already studies on tumor nanozymes in the form of abstracts, case reports, and letters (these studies were excluded due to not meeting the inclusion criteria). This broader time frame helps us identify the technological evolution path and knowledge accumulation process of nanozyme research, thereby enabling a more accurate analysis of the field’s development trajectory. Although there were relatively few directly relevant studies before 2007, this search strategy ensured that we did not miss any early studies that might have had a significant impact on the development of nanozymes, providing a more complete scientific background and historical perspective for our systematic review.

This article comprehensively summarizes the research progress of nanozymes in the field of tumors, including both malignant and benign tumors. During a systematic review of this field, it was found that the application of nanozyme technology in tumor diagnosis and treatment is not limited to malignant tumors but also encompasses benign tumors, thereby reflecting their comprehensive value. In terms of diagnosis, nanozymes assist in differentiating between benign and malignant tumors by sensitively detecting universal biomarkers (e.g., proteins, miRNAs).^[110,111]^ In terms of treatment, their microenvironment-responsive mechanisms (e.g., redox catalysis^[112,113]^) can be adapted for benign lesions requiring intervention.^[114,115]^ Future research should strengthen the validation of benign tumor models and develop novel nanozymes driven by biomarkers, thereby promoting the expansion of tumor diagnosis and treatment from a focus on malignancy to the entire spectrum, ultimately achieving broad coverage and clinical translation of precision medicine.

The current analysis based on the collaboration network also has certain limitations and fails to fully reflect the actual challenges and controversies faced by the research field, especially regarding the barriers to the clinical translation of tumor nanozymes. Firstly, in terms of biocompatibility and safety, nanozyme materials may trigger immune responses, inflammatory reactions, or long-term toxicity in vivo. Some materials (such as metal nanoparticles) have issues with poor biodegradability and unclear metabolic pathways. For example, toxic reactions observed in animal experiments (such as liver and spleen accumulation, oxidative stress damage) are difficult to directly extrapolate to humans.^[116]^ The lack of long-term safety data limits clinical application. Secondly, nanozymes synthesized in laboratories struggle with standardization and large-scale production, showing poor consistency between batches. The absence of unified quality evaluation standards (such as particle size, surface modification, enzyme activity stability) hinders large-scale production and quality control.^[117]^ Additionally, the tumor targeting and delivery efficiency are limited: nanozymes are easily cleared by the reticuloendothelial system in vivo, resulting in low tumor targeting efficiency. The high interstitial pressure and dense matrix of solid tumors obstruct nanozyme penetration. For instance, only 1% to 2% of the injected dose reaches the tumor site; non-targeted distribution leads to damage to normal tissues (such as liver and kidney toxicity).^[118]^ Finally, while nanozymes show promising results in vitro, their activity may decrease in the complex in vivo environment, such as the heterogeneity and hypoxic conditions of the TME. Single functionalities (e.g., only diagnostic or therapeutic) are insufficient to meet the comprehensive clinical needs. For instance, there are significant physiological differences between animal models and human tumors; preclinical efficacy indicators (such as tumor shrinkage rate) may not adequately correlate with patient survival benefits.^[119,120]^ In summary, the clinical translation of nanozymes requires multidisciplinary collaboration (including materials science, biology, clinical medicine, and engineering). Through a closed-loop research approach of “design–optimization–verification–transformation,” the aforementioned barriers can be gradually addressed. In the short term, focus can be placed on local treatments (such as intratumoral injection, intracavitary administration) to reduce systemic risks. In the long term, personalized nanozyme design should be promoted, combined with biomarker screening to identify patients who are most likely to benefit, ultimately achieving the leap from laboratory to bedside. Through multidisciplinary collaboration and systematic research, nanozymes hold the potential to become a significant tool in the field of tumor diagnosis and treatment, providing patients with more precise and efficient therapeutic options.

A limitation of the methodological approach in this study is the reliance on a single database, WoSCC, for literature retrieval, which may not have comprehensively covered relevant research articles included in other important databases such as PubMed and Scopus. Different databases have their own journal coverage and indexing strategies, and this limitation of the retrieval strategy may have led to the omission of some valuable studies, potentially affecting the integrity and representativeness of the research conclusions. Nevertheless, considering WoSCC’s high authority and representativeness as a core database for multidisciplinary research, and the strict inclusion and exclusion criteria and systematic literature screening process we employed, we mitigated the impact of this limitation to some extent. Future research should consider adopting a cross-database retrieval strategy and combining manual searches of important journals and reference tracing to obtain more comprehensive research evidence, enhancing the reliability and generalizability of the findings.

## 5. Limitations

This study conducted a comprehensive bibliometric examination of the field of tumor nanozyme research, including an assessment of its overall scope, progress, notable contributions, and emerging trends. It is recommended that researchers prioritize recent and highly cited literature and focus on topics of significant importance within the field. However, it is important to acknowledge certain inherent limitations associated with this bibliometric analysis. The exclusion of recently published articles may be due to the time lag in data collection. Furthermore, our analysis is limited to articles only from the WoSCC database, which may restrict the breadth of our research findings. Additionally, due to the nature of bibliometric research, we are unable to provide traditional statistical validation (such as confidence intervals, significance testing, etc), which may affect the interpretability of some comparative results. Lastly, although the algorithms used for analysis are objective, we found inherent subjectivity in the interpretation of the data.

## 6. Conclusion

This study represents the 1st comprehensive analysis of the research status and trends of nanozymes in oncology since the turn of the 21st century, utilizing bibliometric approaches. As a novel class of artificial enzyme-like nanomaterials, nanozymes have demonstrated significant promise in the field of tumor diagnostics and therapeutics.

In diagnostic applications, nanozymes replicate the catalytic properties of natural enzymes, allowing for the sensitive detection and imaging of tumor markers, thereby aiding in the differential diagnosis of benign versus malignant tumors. In the therapeutic realm, nanozymes find broad utility, encompassing modalities such as PDT, PTT, chemodynamic therapy, and immunotherapy. They act by generating high concentrations of reactive oxygen species to directly kill tumor cells while also modulating the TME to enhance anti-tumor effects.

China stands as a global leader in this field, with a significantly higher total citation count and average citations per article than any other nation. The United States and Singapore follow in 2nd and 3rd place, respectively. Together, these 3 countries constitute the innovation engine driving tumor nanozyme research.

Future research directions should prioritize: enhancing international collaboration to foster interdisciplinary integration; developing innovative nanozyme materials, such as SAzymes, to enhance catalytic efficiency and biocompatibility; delving deeper into the potential applications of nanozymes in tumor immunotherapy; and expediting the transition of nanozymes from laboratory research to clinical practice. As these research efforts progress, nanozymes are poised to play an increasingly pivotal role in precision tumor care, greatly contributing to the advancement of human health.

## Acknowledgments

We highly appreciated Prof Sanmao Liu for their erudite guidance and persistent encouragement.

## Author contributions

**Conceptualization:** Sanmao Liu.

**Data curation:** Sanmao Liu.

**Formal analysis:** Qiong He, Xuejin Hu, Sanmao Liu.

**Funding acquisition:** Sanmao Liu.

**Investigation:** Qiong He, Sanmao Liu.

**Methodology:** Qiong He, Sanmao Liu.

**Project administration:** Sanmao Liu.

**Resources:** Sanmao Liu.

**Software:** Xuejin Hu, Sanmao Liu.

**Supervision:** Xuejin Hu, Sanmao Liu.

**Validation:** Qiong He, Xuejin Hu, Sanmao Liu.

**Visualization:** Sanmao Liu.

**Writing – original draft:** Sanmao Liu.

**Writing – review & editing:** Sanmao Liu.
